# Surgery of skeletal metastases in 306 patients with prostate cancer: Indications, complications, and survival

**DOI:** 10.3109/17453674.2011.645197

**Published:** 2012-02-08

**Authors:** Rüdiger J Weiss, Jonathan A Forsberg, Rikard Wedin

**Affiliations:** Department of Molecular Medicine and Surgery, Section of Orthopaedics and Sports Medicine, Karolinska University Hospital, Karolinska Institutet, Stockholm, Sweden

## Abstract

**Purpose:**

Skeletal metastases are common in patients with prostate cancer, and they can be a source of considerable morbidity. We analyzed patient survival after surgery for skeletal metastases and identified risk factors for reoperation and complications.

**Patients and methods:**

This study included 306 patients with prostate cancer operated for skeletal metastases during 1989–2010. Kaplan-Meier analysis was used to calculate survival. Cox multiple regression analysis was performed to study risk factors, and results were expressed as hazard ratios (HRs).

**Results:**

The median age at surgery was 72 (49–94) years. The median survival after surgery was 0.5 (0–16) years. The cumulative 1-, 2-, and 3-year survival after surgery was 29% (95% CI: 24–34), 14% (10–18), and 8% (5–11). Age over 70 years (HR 1.4), generalized metastases (HR 2.4), and multiple skeletal metastases (HR 2.3) resulted in an increased risk of death after surgery. Patients with lesions in the humerus (HR 0.6) had a lower death rate. The reoperation rate was 9% (n = 31). The reasons for reoperation were deep wound infection (n = 10), hematoma (n = 7), material (implant) failure (n = 3), wound dehiscence (n = 3), increasing neurological symptoms (n = 2), prosthetic dislocation (n = 2), and others (n = 4).

**Interpretation:**

This study involves the largest reported cohort of patients operated for skeletal lesions from prostate cancer. Our survival data and analysis of predictors for survival help to set appropriate expectations for the patients, families, and medical staff.

Advances in the treatment of prostate cancer have extended life expectancy (Berruti et al. 2000, Carlin and Andriole 2000, Saad et al. 2006). Approximately 70% of the patients with advanced disease can be expected to develop skeletal metastases (Coleman 2001).

The role of orthopedic surgery in patients with skeletal metastases is to treat spinal cord compression and existing or impending pathological fractures in an effort to relieve pain and restore function. Information on outcomes following surgery for skeletal metastases is important for the patients involved, for their families, and for treating physicians (Wedin et al. 2005, Forsberg et al. 2011).

Skeletal metastases from other malignancies are most often osteolytic whereas skeletal metastases from prostate cancer are most often osteoblastic, which may mean unique treatment considerations. However, little attention has been paid to survival and postoperative complications in patients with metastatic prostate lesions.

We have determined patient survival following surgery for symptomatic skeletal metastases in a large cohort of prostate cancer patients. A secondary aim was to identify patient-related and procedure-related risk factors for complications and reoperation.

## Patients and methods

This study involved a consecutive series of patients with prostate cancer who were operated for skeletal metastases from 1989 through 2010. Only patients who had their primary operation at our hospital were included in the study. All data are based on the Karolinska Skeletal Metastasis Register (Wedin and Bauer 2005). This quality-control database prospectively collects individual-based information for cancer patients admitted to the Karolinska University Hospital in Stockholm. The criterion for inclusion is surgical treatment of skeletal metastases. Data on patient identity, age, sex, primary tumor, location of metastases, type of metastases (single skeletal, multiple skeletal, or generalized), surgical procedures (method of fixation and type of implant), and postoperative complications are registered. Generalized metastases are defined as skeletal metastases in combination with visceral metastases. Pathological fractures were defined as skeletal metastases resulting in a dislocation (kyphosis or loss of height for vertebral fractures).

Neurological function in patients with spinal metastases was assessed by the Frankel classification of motor and sensory compromise. It is standard for the neurological function to be assessed preoperatively and within 2 weeks postoperatively. Data for the Frankel classification were collected retrospectively from medical records. Postoperative radiotherapy was offered routinely to all patients. 114 patients in the present analysis were included in a previous publication from our institution (Jansson and Bauer 2006).

### Statistics

Continuous descriptive statistics used median values and ranges. Kaplan-Meier analysis was used to construct the cumulative survival with 95% confidence intervals (CIs) after surgery for skeletal metastases. If patients had more than 1 surgical procedure, only the first operation was accounted for in the survival analysis.

Cox multiple regression was used to study risk factors for (1) death, (2) any complication, and (3) reoperation. The results were expressed as hazard ratios (HRs) with corresponding CIs. If a HR is > 1, the patients at risk are dying at a faster rate or have a higher risk of complications than the patients in the reference group. The assumption of proportional hazards was investigated using graphs of the log-minus-log survivor function against log t over grouped values of the covariates. No signs of insufficient proportionality were detected in the hazard functions, and the log-log plots ran parallel for all covariates. Crude risk factors studied in the simple Cox model were age, anatomical location of the metastasis, type of metastasis (single skeletal, multiple skeletal, or generalized), and pathological fracture. All variables were adjusted for in the multiple Cox model. Wilcoxon's signed ranks test was used to compare preoperative and postoperative neurological function in patients with spine metastases. The level of significance was set at p ≤ 0.05. All statistical analyses were performed using the PASW statistics package version 18.

## Results

We identified 306 patients who met the inclusion criteria; these patients underwent 358 surgical procedures. No patients were excluded from analysis. 16% of the patients had more than 1 site of surgery. The median age at surgery was 72 (49–94) years. Most subjects (62%) were aged 70 years or older. At surgery, most patients had multiple skeletal metastases (73%) followed by generalized metastases (20%) (Table 1).

**Table 1.** Baseline characteristics of study patients at first surgery

	No.
Patients	306
1 operation	258 (84%)
2 operations	44 (15%)
3 operations	4 (1%)
Median age	72 (49–94)
Age group	
< 60 years	27 (9%)
60–69 years	90 (29%)
> 70 years	189 (62%)
Metastases	
Single skeletal	20 (7%)
Multiple skeletal	224 (73%)
Generalized	62 (20%)
Pathological fractures	
Spinal	
Yes	80 (45%)
No	96 (55%)
Non-spinal	
Yes	123 (95%)
No	7 (5%)

**Figure 1.**
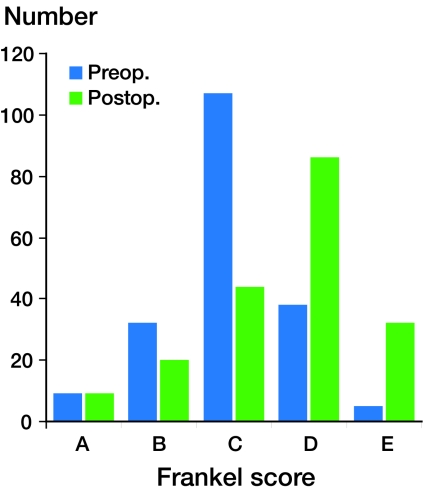
Pre- and postoperative neurological function in 191 patients with spinal metastases, graded according to Frankel (A = complete paraplegia, B = no motor function, C = motor function useless, D = slight motor deficit, and E = no motor deficit).

54% of the skeletal lesions were situated in the spine, followed by the femur (30%), the humerus (8%), and the pelvis (8%) (Table 2). Approximately half of the patients operated in the spine had a vertebral pathological fracture. The main indication for spinal operations was motor weakness due to spinal cord compression (97%); 5 patients were operated on because of painful instability. Regarding surgical procedures for spinal metastases, 58% of the cases underwent decompression in combination with stabilization (Table 2). The overall neurological function in patients with vertebral metastases improved after surgery (p < 0.001). Most cases (55%) improved at least 1 Frankel grade, 39% maintained their neurological function, and 6% deteriorated (Figure 1). Preoperatively, 75% of the patients were non-ambulatory (Frankel A, B, or C) and 25% were ambulatory (Frankel D or E). Postoperatively, 38% of the spine patients were non-ambulatory and 62% were ambulatory.

**Table 2.** Anatomical locations and surgical procedures

	No.
Anatomical locations	358
Spine	193 (54%)
Cervical	1
Thoracic	165
Lumbar	27
Femur	106 (30%)
Femoral neck	37
Trochanteric	27
Subtrochanteric	33
Diaphysis	9
Humerus	28 (8%)
Proximal	9
Diaphysis	17
Distal	2
Pelvis	28 (8%)
Tibia	2 (1%)
Radius	1 (0%)
Surgical procedures	358
Spinal	
Decompression	70 (36%)
Decompression + bone cement	11 (6%)
Decompression + stabilization	112 (58%)
Non-spinal	
Prosthesis	95 (58%)
Osteosynthesis	66 (40%)
Other	4 (2%)
Bone cement	
Yes	125 (35%)
No	233 (65%)
Curetage	
Yes	26 (7%)
No	332 (93%)

Non-spinal surgical procedures were most often implantation of joint prostheses (58%) followed by internal fixation with an internal-fixation device (40%). Most subjects had pathological fractures (95%), i.e. prophylactic surgery for impending fractures was only performed in 5% of the non-spinal cases (Table 1).

### Survival

**Figure 2.**
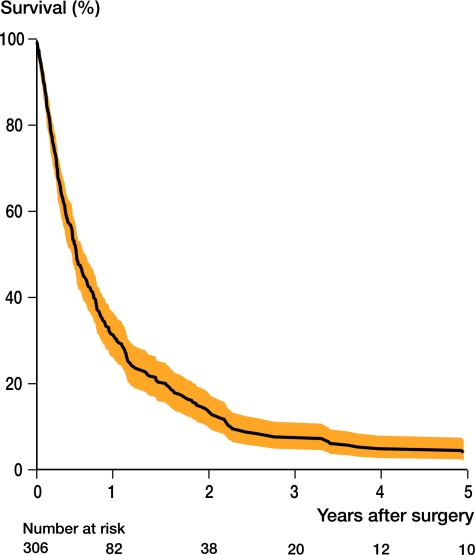
Cumulative survival (with 95% CI) of 306 prostate cancer patients after surgery for skeletal metastases.

The median survival time for the entire cohort after the first surgical procedure was 0.5 (0–16) years. The median survival time after surgery for patients with spinal metastases was 0.5 (0–16) years and for non-spinal metastases it was 0.5 (0–9) years. At the end of the study period, 94% of the patients had died. The cumulative 1- and 2-year survival after surgery was 29% (CI: 24–34) and 14% (CI: 10–18) (Figure 2).

Simple Cox regression analysis revealed an increased risk of death after surgery for patients with lesions in the femur (HR 1.3), pathological fractures (HR 1.3), generalized metastases (HR 2.1), and multiple skeletal metastases (2.2). The corresponding multiple analysis revealed that older age (HR 1.4), generalized metastases (HR 2.4), and multiple skeletal metastases (HR 2.3) were significantly associated with an increased risk of death, while patients with lesions in the humerus (HR 0.7) had a significantly lower death rate (Table 3). We could not detect any predictor for reoperations. However, pelvic lesions (HR 2.3) were risk factors for any complication in both analyses (Table 3). Any complication was defined as either a reoperation or a complication that was treated non-surgically.

**Table 3.** Risk of (1) death and (2) any complication after surgery for skeletal metastases in 306 patients with prostate cancer

	Simple Cox regression **^a^**			Multiple Cox regression **^b^**		
*Endpoint*	HR	(95% CI)	p-value	HR	(95% CI)	p-value
*Death (1)*						
Age						
> 70 years	1.3	(1.0–1.6)	0.07	1.4	(1.1–1.9)	0.01
< 70 years	Ref.			Ref.		
Anatomical location						
Femur	1.3	(1.0–1.7)	0.05	1.2	(0.9–1.6)	0.4
Humerus	0.7	(0.4–1.1)	0.09	0.6	(0.4–1.0)	0.05
All other	Ref.			Ref.		
Type of fracture						
Pathological	1.3	(1.0–1.7)	0.04	1.3	(1.0–1.8)	0.06
Impending	Ref.			Ref.		
Metastases						
Generalized	2.1	(1.2–3.6)	0.006	2.4	(1.4–4.2)	0.002
Multiple skeletal	2.2	(1.3–3.6)	0.002	2.3	(1.4–3.8)	0.001
Single skeletal	Ref.			Ref.		
*Any complication*(*2)*						
Anatomical location						
Pelvis	2.3	(1.3–4.3)	0.007	2.3	(1.2–4.2)	0.01
All other	Ref.			Ref.		

**^a^**Crude HR. **^b^** Adjusted HR. HR: hazard ratio; CI: confidence interval.

### Reoperations and complications

The reoperation rate was 9% (n = 31). The median time to reoperation was 0.5 (0–24) months (Table 4). Complications that were treated non-surgically occurred in 15% of the cases. The median time from surgery to a non-surgically treated complication was 0.2 (0–10) months (Table 5). The overall complication rate (for any complication) was 24%. 

**Table 4.** Reoperations (n = 31)

Reason for reoperation	No., primary metastasis	Median time to reoperation (months)	Treatment
Deep wound infection	5, spine		
	3, pelvis		
	2, femur	1.3	Wound revision
Hematoma	7, spine	0	Drainage
Material failure	3, femur	2.5	Total joint replacement
Wound dehiscence	3, spine	1	Secondary wound closure
Increasing neurological			
symptoms	2, spine	0	Extended laminectomy
Prosthetic dislocation	2, pelvis	0.5	Open reduction Extraction of prosthesis
Non-union	1, femur	24	Total joint arthroplasty
Periprosthetic fracture	1, femur	5.6	Osteosynthesis
Poor initial fixation	1, femur	0.5	Osteosynthesis
Technical error	1, spine	0.2	Extended laminectomy

**Table 5.** Complications treated non-surgically (n = 54)

Type of complication	No., primary metastasis	Median time to complication, months
Superficial wound infection	14, spine	
	2, femur	
	1, pelvis	0.2
Prosthetic dislocation	8, femur	
	7, pelvis	
	1, humerus	0.5
Pulmonary embolism	2, spine	
	2, femur	
	1, pelvis	0.1
Pneumonia	4, spine	
	1, femur	0.1
Heart failure	2, spine	
	1, femur	0
Myocardial infarction	1, spine	
	1, pelvis	0
Wound dehiscence	2, spine	1.6
Perioperative hypoxia		
(hemiparesis)	1, femur	0
Radial nerve palsy	1, humerus	0
Stroke	1, spine	0
Deep wound infection	1, femur	0

## Discussion

Decision making regarding management of skeletal metastases is influenced by factors such as expected duration of survival, overall medical condition, rehabilitation potential, and type of operation required. The goal is to relieve pain and improve function for the maximum amount of time. Patients with a short life expectancy may not benefit from surgery due to rapid deterioration of health and difficulties in managing the postoperative rehabilitation. Some authors have argued that a postoperative lifespan of at least 2 months is required for surgery to be beneficial in extremity metastases (Harrington et al. 1976) and a postoperative lifespan of 3–6 months for spinal lesions (Cybulski et al. 1987, Atanasiu et al. 1993, Tomita et al. 1994). However, these time points are highly debated, and the decision to offer surgery remains patient-specific.

The main indication for almost all spinal cases in our study was motor weakness due to spinal cord compression. Hill et al. (1993) described 70 patients with spinal cord compression secondary to breast cancer. The most frequent symptom was motor weakness (96%) followed by pain (94%), sensory disturbance (79%), and sphincter disturbance (61%). More than half of our patients with spinal involvement improved considerably after surgery, i.e. at least 1 Frankel grade, and approximately 40% maintained their neurological function. These results are similar to those from a recent randomized study, which demonstrated that decompressive surgery in combination with radiotherapy in patients with metastatic epidural cord compression was superior to radiotherapy alone (Patchell et al. 2005).

Several studies have identified prognostic clinical variables that may help to identify patients with a limited expected lifespan after surgery due to skeletal metastases from various cancer types. Pathological fractures, visceral metastases, low hemoglobin level, number of skeletal metastases, and lung cancer as the primary tumor are examples of independent negative prognostic factors for postoperative survival (Bauer and Wedin 1995, Hansen et al. 2004, Nathan et al. 2005, Leithner et al. 2008, Forsberg et al. 2011).

Nørgaard et al. (2010) presented survival data on patients with prostate cancer in a large population-based cohort study. 1-year survival was 87% in patients without skeletal metastasis and 47% in those with skeletal metastasis. The combination of bone metastasis and skeletal-related events reduced the 1-year survival rate to 40%. Zaikova et al. (2011) presented survival data on 260 patients with spine metastases from prostate cancer, treated either with radiotherapy or surgery. They found a 1-year survival rate of approximately 35%, which is comparable to our findings.

We identified several predictors of survival. Younger patients had longer survival after surgery, which has also been described in patients after surgery for skeletal lesions from renal cell carcinoma (Utzschneider et al. 2009). Noguchi et al. (2003) showed that the extent of metastatic skeletal lesions (percentage of positive area on a bone scan) is an independent predictor of death from disease in patients with prostate cancer. We found that patients with a solitary bone metastasis (as compared to multiple metastases) had better survival, which has been reported previously (Wedin et al. 1999, Utzschneider et al. 2009). Skeletal lesions in the humerus were also found to be a positive predictor of survival, a finding for which there is no immediate explanation. This could represent selection bias; for example, unstable fractures in the lower limbs may pose such a significant problem for patients and caregivers that surgical stabilization may be appropriate even when patients have a limited expectation of survival. However, most surgeons at our center do not usually stabilize impending pathological humerus fractures in patients with short expected survival, and as a result, such patients were not included in this study.

We have confirmed the findings of other authors that pathological fractures negatively influence survival (Oefelein et al. 2002, Forsberg et al. 2011). However, only 7 of 130 patients with non-spinal metastases underwent prophylactic stabilization. At our institution, surgery is generally recommended for patients with disabling skeletal lesions in addition to those at risk of fracture using generally accepted criteria. Although efforts have been made to better predict fracture sites and avoid an actual fracture (Coleman 2006), it is unclear whether prophylactic surgery itself confers a survival benefit. Prophylactic surgery was performed in only 5% of our cases. Most patients sustained pathological fractures, which tend to occur later in the disease process in a similar fashion to other oncological diagnoses.

It is often more difficult to treat a pathological fracture than a traumatic fracture. Bone-healing is often impaired due to extensive bone destruction, a catabolic state, and the effects of radiotherapy. The observed reoperation rate of 9% is comparable to previously reported surgical failure rates in pathological fractures (Yazawa et al. 1990, Wedin et al. 1999). In addition, patients with metastases confined to bone (without soft tissue extension) had a lower risk of both reoperation and any complication than did those with generalized metastases.

In addition to complications leading to reoperation, one must also consider those treated nonoperatively. Periacetabular lesions requiring a combination of acetabular metal reinforcement ring and hip arthroplasty emerged as an independent predictor for a complication. This is largely due to the relatively high frequency of hip dislocations, which were treated with closed reduction procedures (7 out of 28) in this subgroup.

Limitations of our study included the lack of information regarding whether adjuvant treatment such as androgen suppression, cytotoxic chemotherapy, or radiation therapy was used. However, at our center, adjuvant radiation therapy is generally used in all patients with surgically treated metastatic bone disease, which is (ideally) started 10–14 days after surgery to allow wound healing.

In conclusion, surgery for vertebral metastases may be the best alternative in patients who are expected to live for at least another 2–3 months, especially if surgery is likely to result in a functional improvement. The patient can, for example, avoid being bedridden and perhaps regain/maintain the ability to ambulate independently. We believe that stabilization of long bone fractures is almost always justified unless the patient has reached a terminal stage and death is imminent.
